# Evaluation of collaborative oral health care planning between older adults and personnel from public dental care and municipal care organizations: a study protocol for a cluster-randomized controlled study in Sweden

**DOI:** 10.1186/s13063-025-08753-6

**Published:** 2025-02-18

**Authors:** Jessica Persson Kylén, Sara Björns, Catharina Hägglin, Lisa Bellander, Annsofi Brattbäck Atzori, Sven Persson Kylén, Ann-Christine Baar, Helle Wijk

**Affiliations:** 1 Department of Health Sciences, University West, 461 86 Trollhättan, Sweden; 2 Centre for Gerodontology, Public Dental Service, Region Västra Götaland, 402 33 Gothenburg, Sweden; 3 Department of Cariology, Institute of Odontology, Sahlgrenska Academy, University of Gothenburg, 405 30 Gothenburg, Sweden; 4 Department of Behavioural and Community Dentistry, Institute of Odontology, Sahlgrenska Academy, University of Gothenburg, Gothenburg, 405 30 Sweden; 5 R&D Department, Primary Health Care, Regionhälsan, Region Västra Götaland, Vänersborg, 462 35 Sweden; 6 Centre for Person-Centred Care (GPCC), University of Gothenburg, Gothenburg, 405 30 Sweden; 7 Institute of Health and Care Sciences, Sahlgrenska Academy, Gothenburg University, Box 457, Gothenburg, 405 30 Sweden; 8 Quality and Patient Safety Unit, Sahlgrenska University Hospital of Gothenburg, Gothenburg, 413 45 Sweden; 9 Centre for Healthcare Architecture, CVA, Chalmers University of Technology, Gothenburg, 412 96 Sweden

**Keywords:** Healthy ageing, Integrated healthcare systems, Oral health, Patient-centred care, Work-integrated learning

## Abstract

**Background:**

Patient participation is key in person-centred care, emphasizing individual choices in treatment. Oral health, integral to overall well-being, is sometimes a neglected part of health. This intervention introduces a novel approach to strengthen person-centred care in homecare settings, employing collaborative, interprofessional teamwork and shared documentation across care organizations. This protocol outlines the design of a cluster-randomized controlled trial (RCT) in Sweden, comparing traditional oral assessments with an interorganizational, team-based oral health care planning model facilitated by a shared digital platform for documentation. The overall aim is to evaluate a person-centred interprofessional and interorganizational model for oral health care planning supported by a digital platform to enable healthy ageing.

**Methods/design:**

The intervention, co-designed with older adults, academic institutions, healthcare providers in public dental care, and municipal organizations, will undergo ethical approval. The RCT will randomize older adults, dental hygienists (DHs) and nursing assistants (NAs) into two groups. The intervention group will attend a two-day workshop on a person-centred, three-step team-based model, while the control group will continue using standard procedures. Thereafter, the three-step collaborative model will be compared to standard procedures. Primary outcomes will be measured using the Revised Oral Assessment Guide (ROAG) and the General Oral Health Assessment Index (GOHAI). Secondary outcomes include health economic evaluations, participation rates and quality of care assessments. Qualitative studies from theoretical perspectives of change and learning based on interviews with key stakeholders will be conducted in both the test and control groups.

**Discussion:**

Taking a co-produced approach where theory and practice shape the research iteratively, a person-centred health care planning model supported by a shared digital platform for home settings is evaluated. Anticipated outcomes include improved oral assessments and a deeper understanding of effective person-centred care practices. The co-produced approach of the intervention is also expected to further develop knowledge regarding co-production within domains of healthy ageing from an oral health perspective. As such, the intervention shapes and fosters co-produced person-centred care and healthy ageing.

**Trial registration:**

ClinicalTrials.gov NCT06310798. Registered on 13 March 2024.

## Background

Healthy ageing is defined as ‘the process of developing and maintaining the functional ability that enables wellbeing in older age’ [1] (p. 4). Functional ability includes the ability to learn and participate in making decisions. As people age with multiple chronic diseases, team-based, interprofessional collaboration is considered key for conducting person-centred care in home settings [2]. Collaborative long-term interventions explored in home settings where older adults and dental and nursing staff participate in health care planning are scarce. Oral health is one crucial but often neglected area in home settings [3]. Therefore, there is a need to develop innovative interprofessional workforce models to support healthy ageing from an oral health perspective [4, 5]. This project is a response to this need. The overall research question is: How can a new team-based work model facilitate person-centred collaboration through oral health care planning with older people in home settings?

### Introduction to current practice: dental care remuneration programme in Sweden

In Sweden, long-term care and home settings are typically work domains for municipal care staff (i.e. for example nurses, nursing assistants, physiotherapists and social workers). The municipal organization is mostly financed by Swedish taxes and the care offered for older adults is regulated by health care legislation and social care legislation. The most frail older adults are cared for in ordinary homes and not in nursing homes [6]. In dental care organizations in Sweden, citizens (> 23 years old) pay a large part of the dental care costs themselves but are offered an annual state-funded dental care allowance and there is also high-cost protection. In dental care organizations, professionals like dentists, dental hygienists and dental nurses work at dental clinics, mainly regulated by the dental care legislation. However, in Sweden, a dental care remuneration programme is offered for people in need of extensive long-term care [7]. The goal of the dental care remuneration programme is to promote oral health, quality of life and proper nutrition. The programme offers highly beneficial subsidized dental care and a yearly oral assessment to people in their homes; the care is provided both in long-term care settings and in homes.

In West Sweden, the yearly oral assessments are conducted by dental professionals, mainly dental hygienists (DHs), from the public dental care organization in older adult’s home settings. Approximately 3500 older adults in West Sweden (Region Västra Götaland) received an oral assessment within the dental care remuneration programme in home settings in 2022. A collaborative agreement recommends that the oral assessments, including oral health care planning, should be conducted cross organizations, including both dental and municipal care staff. Also, the oral health care planning should be documented on an oral care card. The oral care card is a paper-based protocol describing the oral status of the person within the dental care remuneration programme as well as listing recommendations for everyday oral care, including different oral care products and procedures. If the person needs dental treatment, that is also documented on the oral care card. However, reports show that municipal care staff (mainly nursing assistants [NAs] rarely participate in the oral assessments, and recommendations documented on the oral care cards are seldom integrated into the home care records [8, 9]. To our knowledge, there are no studies exploring the change and learning aspects of the interprofessional co-creation of oral health care plans in home settings within the context of a dental care remuneration programme.

### Oral health amongst older adults

Oral health is essential for quality of life [10, 11]. It affects the physical, social and mental well-being of people and communities [12]. Oral health enables vital daily functions, such as the ability to speak, smile, taste, chew and swallow. Older adults who have become frail generally have poor oral health [13–15]. Poor oral health has been associated with malnutrition [16], diabetes [17], aspiration pneumonia [18], and cardiovascular diseases [19]. Like most non-communicable diseases, oral conditions are chronic and socially patterned [20], indicating the need for collaboration between dental care and other healthcare organizations to enable person-centred care and healthy ageing. The fact that older adults often lose contact with dental care with increasing age [21] also supports this statement. Further, recent research shows that attitudes, knowledge and routines concerning oral health and oral care for older adults could be improved [3, 22–24].

### Shared decision-making in the context of healthy ageing

There is a nationwide call in Sweden for the provision of more person-centred and integrated care. The call has a strong base in national legislation [25] and is consistent with international guidelines and research [4, 26] that emphasize person-centred care, such as patient participation in decision-making regarding their care.

Shared decision-making (SDM) is described as a process in which a choice is made by the patient, significant others or both, along with one or more healthcare professionals [27, 28]. It has been described as the crux of person-centred care [29] and has been widely used in healthcare. When patients are involved in SDM, they experience more recognition and gain more knowledge regarding the decisions made [30]. SDM is initiated when difficult choices must be made amongst several options with several potential outcomes, which might be regarded differently from person to person. For example, in the context of oral health care planning in a home, it could be important to plan, in a methodological way, whether a person wants to receive assisted oral care. Also, if there is a need for dental treatment or follow-up, whether they prefer to schedule an appointment at home or at the dental care clinic. Through SDM, someone with a decisional conflict can be assisted in making a quality decision based on the best available evidence about the risks and benefits of options and what matters most to the person [31].

### Use of digital tools across organizational boundaries, facilitating person-centred, integrated care

In-home care, SDM is facilitated when health professionals work in teams and have access to common tools [32]. Several studies suggest that digital tools can contribute to enabling person-centred care [33–35]. Digital tools are seen as the intermediaries amongst healthcare professionals and older adults. However, professionals within diverse organizations do not have access to all information concerning the health status of older adults [36]. This might contribute to older adults receiving conflicting information from different care personnel. Also, different care professionals tend to focus on their own fields of expertise [37]. This implies that it is not enough to use digital tools to connect older adults with their care providers, but that it is also critical to link their care providers together as older adults inevitably move between them.

The transitions between care providers, especially those from different organizations, have been critical points of failure which have led to undesirable health outcomes [34]. Patients affected by such a lack of coordination have reported feelings of being lost in the system, frustration, disempowerment, and lack of trust [38]. The possibility to conduct integrated care, to coordinate and communicate amongst professionals within diverse professions and organizations involved in the same care processes, is often lacking [37, 39]. This results in fragmented care [39, 40].

Consequently, professionals might regard an older adult’s diagnosis and treatment without considering their relevant medical history or being able to acknowledge the complexity of the whole person during health care planning. If older adults’ health information is accessible, it becomes possible to personalize the care pathway for each individual, contributing to person-centred care. Therefore, the overall aim of this project is to evaluate a person-centred interprofessional and interorganizational model for oral health care planning supported by a digital platform to facilitate healthy ageing.

## Theoretical framework

**Fig. 1:**
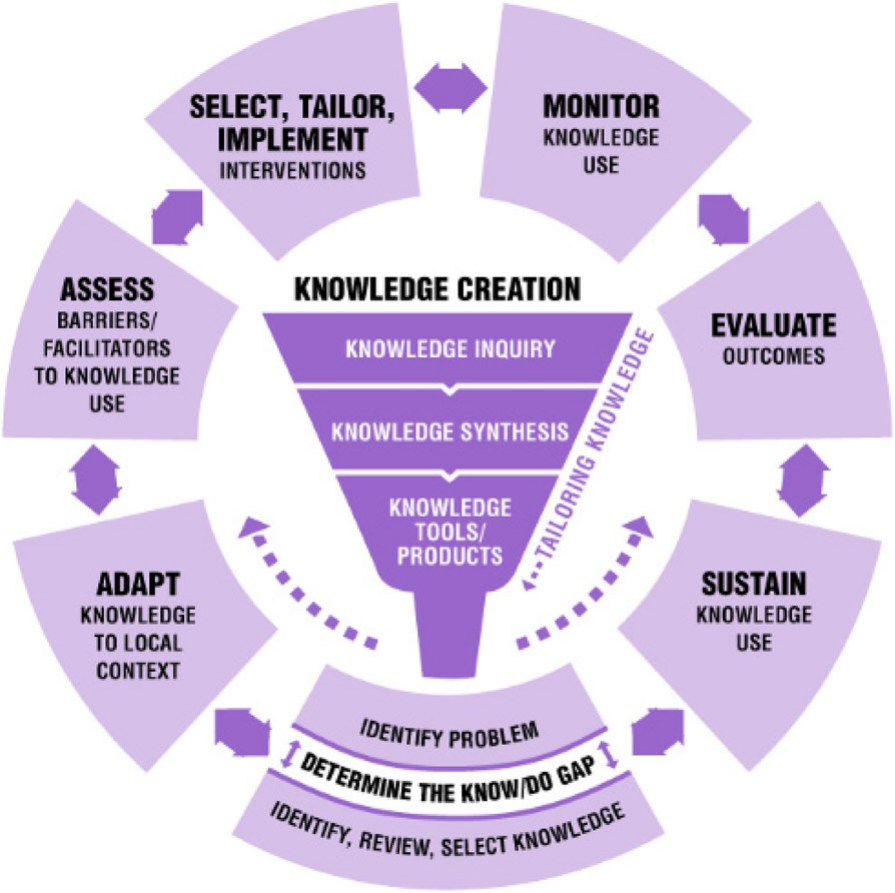
Knowledge To Action (KTA) framework according to Graham et al. [41]

The Knowledge To Action framework (KTA framework) [41] has been widely used within healthcare domains to assist innovative research projects being implemented into practice in a successful way. The KTA framework has two main components, knowledge creation and an action cycle, and it focuses on seven phases for the application of knowledge in practice settings (Fig. 1; Table 1).

**Table 1:** Summary of the intervention, guided by the KTA framework [41]

Phase	Action	Outcome
I. Identifying the problem or gap that needs attention, and identifying, reviewing and selecting the knowledge that can solve that problem or gap	Through an interprofessional infrastructure between dental care and municipal care [9, 42], it was concluded that oral care cards were difficult to use in regard to coordination, patient safety, communication and ethical considerations	✔ The oral assessments within the dental care remuneration programme model and the oral care cards were tentatively revised into a team-based work-model based on learning and guiding each other ✔ A paper-based prototype for a digital interorganizational oral care card was created
II. Adapting or tailoring the knowledge to the local context	The new team-based work-model and oral care card prototype were tested [43, 44] in 24 ordinary homes of older adults [45]	Data from oral assessments were analysed through: ✔ Decisional needs assessment during interprofessional oral health planning [45]
III. Assessing barriers to and facilitators of knowledge use	Researcher (JPK) participated in a course at University of Ottawa, Canada, in clinical decision-making Systematic search, review and theory analysis regarding SDM in oral health domain [46]	✔ Common theories regarding interprofessional decision-making between dental care and healthcare were concluded to be lacking Knowledge and tools for decision-making during oral health care planning were integrated into the oral care card prototype
IV. Selecting, tailoring and implementing the interventions	Development of a digital prototype as a shared tool for the team-based model. The work-model and tool were co-produced and tested in three (SB, ABA, JPK) homes of older adults Five days of fieldwork within the dental care remuneration programme (JPK)	✔ The future digital tool for oral assessments could be designed as a digital platform since it needs to be accessible and integrated in several organizations and systems ✔ Dental visits could be offered as both regular, at clinic, and in-home settings, through a mobile dental unit. The ability to schedule these alternatives should be integrated within the digital platform ✔ The entire process, across organizations, within the dental care remuneration programme was plotted ✔ Formation of a steering committee for implementation in several organizations
V. Monitoring the knowledge use	Formation and establishment of a sister project, a collaborative project for innovations focusing on organizational aspects (public dental and municipal care organizations) when developing the team-based work-model and the digital platform	✔ Through the sister project (the collaborative project for innovations), the new team-based work-model and digital platform can be further developed in regard to organizational needs, for example, from legal and engineering perspectives ✔ Developing a method, not only a model and digital tool
VI. Evaluating outcomes	Evaluation of method, model and digital tool	**✔ Randomized controlled trial** ✔ Qualitative results ✔ Health economic evaluation
VII. Sustaining knowledge use	Through the sister project, organizational ownership, responsibilities, and processes are mapped and discussed. Key stakeholders are included in every step of the projects, including older adults	✔ A new interprofessional, team-based method, work-model and digital platform aiming at healthy ageing

## Methods

### Procedure of intervention

From a theoretical understanding of interventions as depending on theory and practice, this intervention is inspired by Schön [47], who states that stakeholders, through reflection-in-action, contribute to both theory and practice in an iterative way. Therefore, the empirical base for this research is important, and stakeholders from multiple domains are included and invited to co-create and participate. Guided by the KTA framework [41], the first four phases describe actions that have already been conducted, while the three last action phases describe planned actions, evaluations and a plan for implementation. The randomized controlled trials are described in the sixth phase.

#### Phase I: Identifying the problem or gap that needs attention, and identifying, reviewing and selecting the knowledge that can solve that problem or gap

Within a collaborative project between dental and municipal care organizations (in Swedish; “the TAIK-project”), an interprofessional infrastructure was established for learning at work and being able to conduct joint oral health care planning, and to perform and follow up activities on an interorganizational level facilitated healthy ageing [8, 42, 48]. It was concluded that oral care cards were difficult to use within the dental care remuneration programme and that patient safety needed improvement [8, 9].

Two hypotheses were suggested:

I. The dental care remuneration programme work-model could gain from including older adults and the NAs further in the oral assessments to facilitate person-centred, integrated care and healthy ageing, i.e. through collaborative learning and decision-making [49].
II. The oral assessments would gain from being based on common validated instruments and shared digital tools, which could facilitate healthy ageing and improve the quality of care [32].

From the two hypotheses, the model for oral assessments within the dental care remuneration programme was tentatively revised to:

I. Aspects of self-reported health, including oral health, by the older adults.
II. The Revised Oral Assessments Guide (ROAG) [50] will be used to facilitate a learning environment (ROAG performed in collaboration between older adults, DHs and nursing staff). ROAG is a validated instrument used within municipal home healthcare by nurses and NAs. It was also included to provide a common language. When ROAG indicated difficulties regarding oral hygiene (= score 2 or 3 on ‘teeth’ or ‘gum’ within ROAG), the cause of the inability to manage oral hygiene tasks could be explored inspired by the Oral Hygiene Ability Instrument (OHAI) [51].
III. Health care planning should be theoretically anchored, supported by the Ottawa shared decision-making framework [52], a well-cited theoretical framework which also includes the surrounding for decision-making, except for the person making the decision.

#### Phase II: Adapting or tailoring the knowledge to the local context

The new work-model and the prototype were tested in 24 interprofessional and interorganizational teams taking a socio-cultural approach [43, 44] in home settings in October–November 2022 in West Sweden. The work-model for the teams (1 older adult, 1 DH, 1 nurse) was supported by a digital prototype (paper-based mock-up) for oral assessments. In total 24 older adults, seven DHs and eight registered nurses in home healthcare participated [45].

#### Phase III: Assessing barriers to and facilitators of knowledge use

The testing of the work-model and digital prototype revealed that the collaborative decision-making phase of interprofessional oral health care planning was challenging. Therefore, one researcher within the project (JPK) participated in a course in clinical decision-making at the University of Ottawa. To better anchor the patient participation approach during decision-making in the oral assessments in the dental care remuneration programme, plans were made to integrate the Ottawa decision support framework into a new team-based work-model supported by a digital platform. Since the present documentation within the dental care remuneration programme revealed a lack of evidence-based recommendations, the planned digital tool will support and suggest evidence-based treatments derived from oral assessments at the individual level guided by Sweden’s national dental guidelines [51]. Complying with these suggestions will not be mandatory.

The participants in the 24 teams in home settings requested options for dental care visits—(i) mobile units in home settings, (ii) digital consultation or (iii) regular visits at the clinic. These options should be integrated into a future shared digital tool to facilitate better coordination. Also, the participants asked for options regarding assistance for oral care (i.e. reminders, assistance with the distribution of oral hygiene products or with the entire process of oral hygiene) to be inserted into a future digital tool.

#### Phase IV: Selecting, tailoring, and implementing the interventions

In June 2023 a second digital prototype for the digital platform was initiated. Based on previous knowledge, the platform was suggested to enable several stakeholders (such as older adults, DHs, nurses, NAs, management and relatives of older adults) to have access to tailored information regarding oral assessments. Three teams in home settings (participants = older adults, DH and nurse) further tested the work-model and the digital prototype in September 2023 (SB, ABA, JPK). One member of the research team (JPK) also conducted fieldwork during five workdays between October 2023 and March 2024 within the present dental care remuneration programme. The fieldwork focused on overall care processes and on barriers to—and facilitators of—healthy ageing. The option to schedule a mobile unit to provide dental care in home settings was integrated into the digital platform.

#### Phase V: Monitoring the knowledge use

Further development of the digital platform is planned for 2024 within a collaborative sister project for innovation. For example, a lawyer will assist in enabling interorganizational documentation between dental care and municipal organizations. Also, a steering committee and a project group will plan and monitor the process of development and implementation.

#### Phase VI: Evaluating outcomes 

**Fig. 2:**
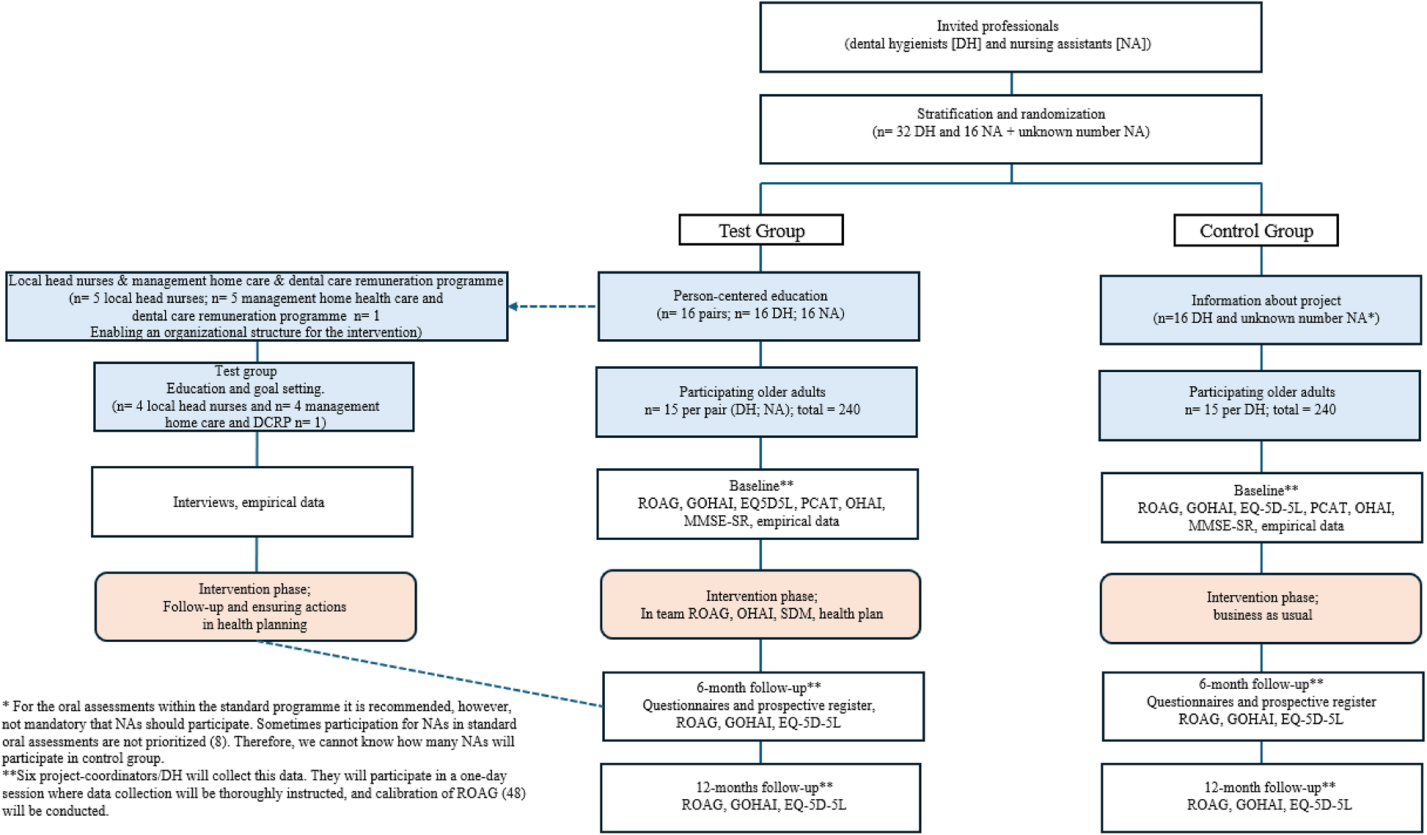
Procedure of RCT

The evaluation of the revised team-based work-model assisted by a digital platform is planned to be conducted as a cluster-randomized controlled trial (RCT) (Fig. 2).

*The control group* will conduct oral assessments as per standard praxis: DHs, preferably in pair with a NA, perform an oral assessment on an older adult in home settings. Instruments used will be a mouth mirror, torch and oral care card—a paper where oral status and recommendations are documented. The following aspects are to be documented, visible caries or periodontitis, xerostomia, the oral hygiene products that the older adult use, and if the older adult goes on regular dental visits. If the older adult has pain in the mouth this is also documented, and a suggestion for booking time at the dental clinic is then recommended.

*The test group* will do oral assessments in teams of older adults, DH and NA. The following instruments will be used, a mouth mirror, a torch and a shared, digital platform. A three-step model is suggested – where subjective information is first documented in the digital, shared platform. Thereafter, valid instruments for oral assessments, understandable for all (ROAG; 48), will guide the participants. If there are notifications on problems with conducting oral hygiene, the reason for the problem will be investigated through a validated instrument, OHAI [51]. Finally, shared health care planning will be conducted based on the previous findings and the older adults’ preferences. If indicated, dental treatments will be recommended either through an established dental clinic or a mobile dental unit.

**Fig. 3:**
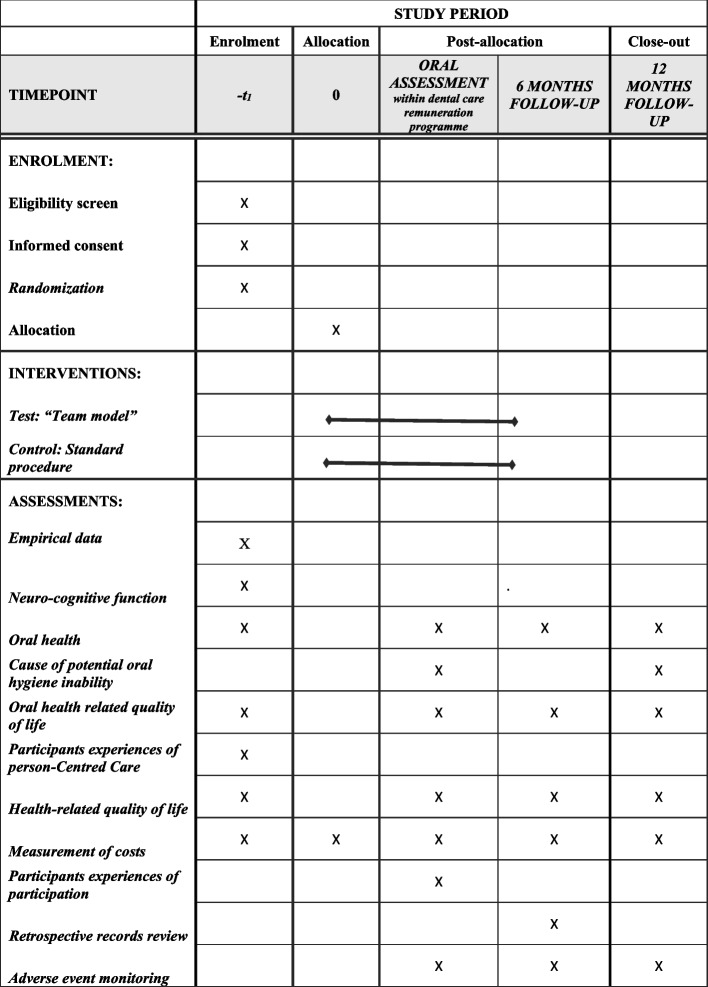
Schedule of enrolment, interventions, and assessments within the intervention

All participation within the RCT, both in terms of professionals and older adults, will be voluntary and the project will be ethically reviewed. In West Sweden, the public dental care organization (including 3200 dental care professionals) is responsible for performing all oral assessments within the dental care remuneration programme. This guarantees a large empirical base. A schedule of enrolment, interventions and assessments according to Standard Protocol Items: Recommendations for Interventional Trials (SPIRIT) reporting guidelines [53] is presented in Fig. 3.

In pairs, DHs within public dental care organizations in West Sweden will be randomized into test and control groups. Due to potential geographical specificities, NAs within the same municipality as the test control DHs will be asked to participate. As such, the test group will consist of 16 DHs and 16 NAs. They will form teams of two, and each team will collaborate with 15 older adults to conduct oral assessments and health care planning in home settings. The control group will consist of 16 DHs where each DH will perform oral assessments according to the dental care remuneration programme – that is, they will conduct business as usual on 15 older adults each. It is not mandatory but recommended for NAs to participate in the oral assessments; therefore, the number of participating NAs within the control group is unknown. Inclusion criteria for the older adults are having home care and being registered for the dental care remuneration programme. Inclusion criteria for the DHs are that they (in addition to their clinical work in public dentistry) perform oral assessments within the dental care remuneration programme, and for the NAs to work in-home care. *Exclusion criteria* for older adults is to be younger than 65 years old. In total, 480 oral assessments and health care planning sessions will be performed within the RCT including 480 older adults, 32 DHs and 16 NAs (+ unknown number of NAs in the control group) (Fig. 2).

The test group will participate in a two-day course, where initially an informed consent form will be signed by all participants, thereafter they will practise:

- Oral assessments according to ROAG [50].
- Oral hygiene ability assessments according to OHAI [51].
- Oral health-related quality of life assessment with the GOHAI [54, 55].
- Shared decision-making according to ODSF [52].
- Using the digital platform.

The course will be both theoretical and practical with elements of reflection. Also, test group management within dental and municipal healthcare will participate in a common half-day course where they will learn about the intervention and the digital platform. A dialogue regarding obstacles and opportunities will facilitate a common understanding and the continued implementation process. They will also set common goals regarding how to support the intervention.

At baseline, six project coordinators (DHs) will collect informed consent forms from all participants, before the intervention. The six project coordinators will collect data and manage spontaneous reports of adverse events and other unintended effects of trial interventions. Empirical data will be collected. All older adults that participate will be enrolled in the dental care remuneration programme and in-home care. Participants within the test and control groups will answer on the following parameters at baseline, which will guide the evaluation.

At baseline:

- Empirical data (participant characteristics, i.e. sex and age).
- Mini mental state examination (MMSE-SR) [56].
- ROAG [50].
- GOHAI [54, 55].

*Primary outcomes* -* change from baseline to 12 months follow-up:*

- Older adults’ oral health status according to ROAG (50).
- Older adults’ self-perception of their oral health-related quality of life according to GOHAI [54, 55].

*Secondary outcomes*

- SURE test [57] before and after health planning in oral assessments. The SURE test is based on four validated questions which describe the older adults’ uncertainty about which treatment to choose and aspects contributing to uncertainty about the benefits and risks of each option, what matters most to the older adults, support in making a choice and feeling sure about the best choice.
- Person-Centered Care Assessment Tool [58] to measure the professionals’ experience of the extent to which the care is experienced as being person-centred.
- All participants (DHs, NAs and older adults) will be measured by health-related quality of life according to EQ-5D-5L [59].
- OHAI [51], for measuring the ability to perform oral hygiene tasks. The OHAI should be used when indicated, when ROAG [50] shows 2 or 3 at the variables ‘teeth’ and ‘gum’, or if the older adult expresses concerns about difficulties with maintaining oral hygiene.

All scales will be measured in both the test and control groups (relation 1:1). Thereafter, test (n = 16 pairs of DHs and NAs) and control (*n* = 16 DHs and *n* = unknown NAs) groups will conduct 15 oral assessments each in home settings (Fig. 2). The DHs and NAs will individually rate their team performance level after each oral assessment from 1 to 10 (1 = very bad, 10 = excellent). Within the test group, options regarding dental care visits will be (i) regular dental care visits, (ii) digital consultation, (iii) mobile unit at home settings and (iv) doing nothing. Within the control group, the options regarding dental care visits will be ‘business as usual’, namely, (i) regular dental care visits, (ii) digital consultation or (iii) doing nothing.

Follow-up at 6 months:

- Retrospective records review: Number of records including oral health care aspects within municipal care. Number of older adults having established contact with dental care. Number of older adults having been scheduled for dental treatment at mobile dental units and what treatment has been conducted.
- The oral health care recommendations will be analysed and compared to existing evidence-based guidelines and recommendations [60].
- Older adults’ oral health status according to ROAG [50].
- Older adults’ self-perception of oral health according to GOHAI [54, 55].
- All participants’ (DHs, NAs and older adults) health-related quality of life according to EQ-5D-5L [61].

Follow-up at 12 months:

- Oral health status according to ROAG [50].
- Older adults’ self-perception of oral health according to GOHAI [54, 55].
- All participants’ (DHs, NAs and older adults) health-related quality of life according to EQ-5D-5L [61].
- Person-Centered Care Assessment Tool [58].

### Safety

All adverse events during the study will be recorded at the 6- and 12-month follow-up, including severity, duration, and causality (i.e. relation to treatment). There is no anticipated harm and compensation for trial participation. The older adults will be offered treatment according to the standard dental care remuneration programme as a provision for post-trial care.

There will be no special criteria for discontinuing or modifying allocated interventions.

### Data analysis

Descriptive data will be presented using the mean and standard deviation (SD) or median and interquartile range for numeric variables, as appropriate. Categorical variables will be presented in numbers and percentages. The efficacy evaluation will be conducted according to intention-to-treat, including randomized subjects analysed by randomized treatment. Missing data will be imputed using multiple imputation. Drop-outs will be summarized descriptively by the treatment group with at breakdown of reasons for withdrawal.

Statistical analyses will be performed with appropriate methods for cluster RCTs using mixed effects models with DH as a random effect to account for intra-cluster correlations. Numeric variables will be adjusted for baseline values, if applicable. All questionnaire scales will be treated as numeric in this regard. Results will be presented using the mean difference between groups with 95% confidence intervals. The primary endpoints (change in ROAG and GOHAI from baseline to 12-month follow-up) will be tested at significance level *α* = 2.5%, a two-sided test, retaining the type I error rate of the primary endpoints at the 5% level. All other tests will be two-sided and conducted at the 5% significance level. No interim analyses will be conducted.

A total of 32 DH (16 per group) and 480 older adults (240 per group) will be included to demonstrate a clinically meaningful difference of 1 unit in ROAG at 80% power, adjusted for baseline values. The following assumptions were made: SD of change scores from baseline values = 3 since the older adults as a group are heterogeneous [45]. Weak to moderate correlation between baseline values and change scores = 0.3, m = 15 older adults per DH, a weak intraclass correlation coefficient within clusters = 0.05, significance level *α* = 0.025, two-sided test, and 25% drop-out [59]. A weak intraclass correlation coefficient is expected since DH is a licensed profession in Sweden, and ROAG is a validated instrument which shows little intraclass difference over time in nursing care [62]. A blinded evaluation of the SD, correlation, and intraclass coefficient will be conducted when 6-month follow-up data has been retrieved for 50% of the planned study participants, with a possibility for sample size adaption if judged necessary. Sample size calculations were conducted using SAS/STAT® Software, Version 9.4 of the SAS System for Windows (SAS Institute Inc. Cary, NC, USA).

In parallel, research will be conducted to describe the development of the interprofessional and interorganizational digital platform. Also, individual interviews are planned with professionals (DHs, NAs and managers), the dental team of the mobile unit (dentist and dental nurse) and older adults within the test and control groups. Further on, a health economic evaluation will be conducted.

#### Phase VII: Sustaining knowledge use

The close collaboration between researchers and the several organizations involved in the project serves as a good foundation for sustaining knowledge use. The research group consists of key stakeholders, representing older adults, a municipal organization (Trollhättan stad), universities (University West, Gothenburg University and Chalmers), Research & Development office, Region Västra Götaland and Public Dental Care Region Västra Götaland (involving professionals within the dental care remuneration programme). The several knowledge domains represented are considered to approach the complex social dilemmas within the intervention in a multi-phased way. Thus, an iterative way of approaching and integrating theory and practice is expected.

On an individual level, the diverse knowledge domains mentioned are represented by a multidisciplinary research team, including public and patient involvement. The research team is organized as a steering committee (HW, CH, SPK, AB) that is responsible for overall quality and for planning and leading the research. The members represent person-centred care and nursing (HW), gerodontology and dental care (CH), organizational change and psychology (SPK) and lived experiences as an older adult, carer as next-in-kin and national commissioner in the Swedish retirement union (in Swedish Svensk pensionärs förening [SPF seniorerna]; patient representative) (AB). The members of the wider research team (including the steering committee and also JPK, SB, ABA and LB) have experience with work-integrated learning and oral health (JPK), public health and oral health (SB), innovation and municipal organization (ABA; public involvement—representative for the experience of work within municipal home care and the dental remuneration programme) and gerodontology and dental care (LB).

All members within the research group have an educational and practical background as registered or licensed caregivers. This organization, involving several persons having experiences and knowledge within several key areas, is expected to plan to implement and sustain knowledge use in an interactive way. During the RCT a project management group consisting of JPK, SB and ABA will monitor, together with the six coordinators/dental hygienists the data collection. The data monitoring committee is the project management group including the six coordinators. During the phase of the intervention, they will have daily contact. The project management group will meet every week during the period of data collection. Also, they will report a written report to the steering committee, which will have monthly meetings. If indicated, the steering committee will have meetings more often. All members within the research group (steering committee and project management group) will be involved in the training of the test group before the intervention, based on their domain of expertise.

## Discussion

The study helps to fill existing knowledge gaps, and we have summarized the significance and contribution to research as follows:

- Most research concerning the oral status of older adults is based on older adults being able to participate in the data collection at dental care clinics [63]. The digital platform will provide a foundation for a register of the oral status of older adults in home settings, including older adults who are unable to participate in dental care clinics. This will support understanding of oral status in home settings of older adults, and of how to design and plan interventions to support healthy ageing.
- Older adults are included in every part of this project, including as co-authors. This emphasizes the participatory aspect of healthy ageing, and also the notion of conducting research *with*, as a complement to *on* human beings [64]. Stakeholders within municipal organizations are also included as partners, emphasizing the importance of bridging theoretical and practical understanding when developing research. Co-creating knowledge within social practices of working life, might contribute to further understanding lifelong learning and healthy ageing.
- The way in which health care planning is conducted in home settings is crucial for conducting person-centred care [2]. To our understanding, there is no available conceptual model involving older adults as well as dental and healthcare professionals based on a theory of shared decision-making. Within the project, this will be explored and further developed.
- Having a shared tool during health care planning in home settings is important for health care planning and decision-making [32]. There are, to our knowledge, no interorganizational digital platforms for older adults that bridge dental and municipal healthcare and enable long-term, integrated care. Evaluating the interprofessional shared digital platform and the activities within the work-model will further contribute to theory and practice by adding knowledge in these domains.
- Healthcare and dental care need to be further integrated [4] and poor oral health is often preventable, chronic and socially derived [20]. The use of the interorganizational digital platform can probably be extended to other areas besides home settings. This could further enable diverse interorganizational projects and innovations, which might contribute to research closing the gap between the separate knowledge domains of healthcare and dental care.
- The coordination aspects of the interorganizational project are major [9]. Assisted by the digital platform, coordination might be further developed with artificial intelligence (AI). As such, the project may also further contribute to research within artificial intelligence areas since there is a need for bridging the gap between research within the domains of AI design and healthcare [65].
- A prerequisite for healthy ageing is change and learning [49]. How learning and change are defined, discussed, and considered is therefore crucial. For example, implementing a new work- model and a digital platform requires change and learning. However, there is uncertainty about what kinds of work-life experiences generate what kinds of knowledge and actions [66]. Using theories within change and learning when analysing outcomes within this project might contribute to identifying and learning new knowledge [67]. Also, iteratively co-developing the work-model and digital platform is expected to bridge research and practice for the promotion of healthy ageing.

The results of this study can be applied in clinical practice immediately. If the interorganizational model, supported by the digital platform, has positive outcomes, the model and digital platform can readily be implemented in home settings in Sweden, and probably also internationally. Furthermore, the results of the study can guide the development of person-centred care and guide future change and learning approaches within interorganizational collaborative domains to facilitate healthy ageing.

## Trial status

This study is registered at Clinical Trials.gov—registration date 13 March 2024. This is the first study protocol, dated the 25th of November 2024. Recruitment and randomization of participants are planned to start in September 2025. The data collection plan is to be completed in September 2026.

### Project organization

This project is conducted in collaboration with the Center of Gerodontology and the Dental Public Health Care organization Region Västra Götaland; University West, in Trollhättan; University of Gothenburg; Chalmers University of Technology (CUT) – Centre for Healthcare Architecture; in joint discussion with municipal care organizations, older adults and their significant others. In case of changes, we will notify sponsors and funders, and a copy of the revised protocol will be sent to the Investigator Site File. Any deviations from the Protocol will be fully documented using a breach report form. We will also update the protocol in the clinical trial registry.

## Data Availability

Participants within the project have given permission for their data to be used. In agreement with GDPR, the data are to be used only for the purpose of these studies.
